# No Evidence That Baseline Prefrontal Cortical Excitability (3T-MRS) Predicts the Effects of Prefrontal tDCS on WM Performance

**DOI:** 10.3389/fnins.2018.00481

**Published:** 2018-07-17

**Authors:** Lotte J. Talsma, Julia A. Broekhuizen, Job Huisman, Heleen A. Slagter

**Affiliations:** ^1^Department of Psychology, University of Amsterdam, Amsterdam, Netherlands; ^2^Amsterdam Brain and Cognition Center, University of Amsterdam, Amsterdam, Netherlands

**Keywords:** tDCS, MRS, working memory, individual differences, prefrontal cortex, GABA, brain stimulation

## Abstract

Transcranial direct current stimulation (tDCS) over the left dorsolateral prefrontal cortex (lDLPFC) is a promising tool to enhance working memory (WM) in clinical as well as healthy populations. Yet, tDCS does not affect everyone similarly: whereas tDCS improves WM in most individuals, some individuals do not, or actually show detriments in WM performance after stimulation. One hypothesis that has been put forward to account for individual differences in tDCS response is that baseline cortical excitability levels in the stimulated cortex may determine the strength and the direction of the effects of tDCS. Specifically, by locally affecting neuronal excitability, tDCS may interact with baseline cortical excitability levels, thereby pushing or pulling individuals toward or away from an optimal level of cortical functioning. In the current study, we put this hypothesis to the test with regard to prefrontal cortex stimulation and WM. In 20 healthy male participants, using magnetic resonance spectroscopy (MRS) at 3T, we measured concentrations of Glutamate and GABA in the lDLPFC and calculated individual Glutamate/GABA ratios as a measure for cortical excitability. Subsequently, in two stimulation sessions, we once applied anodal and once cathodal tDCS over the lDLPFC (20 min, 1 mA). Stimulation was always applied in the second block of three blocks of a WM updating task. Surprisingly, at the group-level, we found no effects of anodal or cathodal stimulation on WM performance. Yet, in line with previous studies, large individual variability was observed in the strength and direction of tDCS effects; whereas about half of the participants improved, the other half showed lower accuracy after stimulation. This was true for both anodal and cathodal tDCS. Nevertheless, contrary to our expectations, individual baseline prefrontal cortical excitability did not predict these individual differences in the effect of anodal or cathodal stimulation on WM accuracy. Future studies with larger sample sizes, which use higher magnetic field strengths (e.g., 7T) to measure cortical excitability and/or apply individualized stimulation protocols, are necessary to shed more light on the influence of baseline cortical excitability on effects of anodal and cathodal tDCS over lDLPFC on WM performance.

## Introduction

Transcranial direct current stimulation (tDCS) is a non-invasive brain stimulation technique that has rapidly gained scientific interest as a promising tool to enhance cognitive functions, such as working memory (WM). In tDCS, a low-voltage electrical current (typically < 2 mA) is run between two or more electrodes placed over specific brain areas at the scalp. A small portion of this current reaches the brain and influences the membrane potentials of neurons such that they are more (under the anode) or less (under the cathode) prone to fire action potentials (Nitsche et al., 2008; Kuo and Nitsche, 2012). Thus, tDCS can directly modulate neuronal excitability in particular brain regions, thereby affecting brain and cognitive functioning. As such, tDCS may be used as a tool to enhance brain function and cognitive abilities such as WM.

Our WM allows us to maintain and monitor information over brief periods of time (Baddeley et al., 1996) and thus plays a core role in many daily-life situations. One brain region that is critically involved in WM is the left dorsolateral prefrontal cortex (lDLPFC) (Owen et al., 2005). Initial studies with tDCS found that anodal tDCS stimulation over the lDLPFC could improve verbal WM in healthy (Fregni et al., 2005; Ohn et al., 2008; Andrews et al., 2011) and clinical populations (e.g., Boggio et al., 2006), making it a promising method for enhancing WM functioning. However, since those pioneering studies, the effects of tDCS on cognition have been less conclusive (Jacobson et al., 2011), with several studies questioning the ability of anodal lDLPFC stimulation to robustly improve WM performance (see meta-analyses by Bennabi et al., 2014; Brunoni and Vanderhasselt, 2014; Dedoncker et al., 2016; Hill et al., 2016; Mancuso et al., 2016).

Recently, we examined if multiple sessions of anodal tDCS over the lDLPFC during a verbal WM task (a letter N-back updating task) would lead to increasing gains in WM performance across training sessions in healthy adults (Talsma et al., 2016). Replicating previous single session studies, we found that anodal compared to sham tDCS led to an increase in WM performance, but only in the first session. Moreover, we observed that the effects of anodal tDCS were quite variable across individuals in both strength and direction, and that these individual differences in the effect of anodal tDCS during WM training predicted the extent to which individuals performed better on subsequent WM transfer tasks. Looking at the individual subject data, we found that 2 of our 15 subjects in fact showed worse performance after anodal stimulation both on the trained and transfer WM tasks.

Large variation in the effect of tDCS on WM between subjects is problematic with regard to the practical use of tDCS as method to enhance WM function in everybody. Moreover, a better understanding of individual differences in tDCS responsiveness may help resolve current inconsistencies in the literature, as it may explain why overall tDCS effects are found in some groups of subjects, but not in others. Therefore, for the current progression of the tDCS field, investigating the determinants of individual differences in tDCS response is a pivotal scientific direction to explore.

In recent years, several possible explanations have been proposed for the relative large variability in tDCS response. Currently, many of these are directed at the question whether the admitted current may in fact reach the target brain area in all subjects. Modeling studies have indicated that tDCS current flow with conventional standard tDCS set-ups can be strongly affected by individual differences in anatomy, skull thickness and folding of the cortex (Opitz et al., 2015). Another proposal that has been put forward to account for inter-individual differences in tDCS response is that tDCS effects may depend on baseline functioning of the stimulated area. Specifically, it has been proposed that prefrontal tDCS may enhance WM performance only in subjects in which the prefrontal cortex is in fact engaged in the task, assuming that tDCS needs some baseline activation to ‘grasp’ onto (Berryhill and Jones, 2012). Postulated more broadly, the effect of tDCS may depend on the way in which a stimulated brain region is currently involved in the task. Namely, when brain region engagement is already optimal, tDCS may cause overstimulation, resulting in worse performance, whereas when brain region engagement is suboptimal, tDCS may optimize brain function, resulting in improved performance.

Recently, Krause et al. (2013) proposed a theoretical model to explain these baseline and tDCS effects interactions at the cellular level. More specifically, they suggested that since the cortical excitability level of a particular brain area critically determines neuronal firing rates, it plays a pivotal role in cortical functioning. In an optimal situation, the cortex is active enough for functional firing to effectively take place, but at the same time inhibited enough to reduce noise and unwanted firing (Turrigiano and Nelson, 2000; Turrigiano and Nelson, 2004). However, both too high and too low excitability may be detrimental for functional performance, and the relation between cortical excitability and performance can thus be described as an inverted U-curve. Depending on an individual’s initial position on the curve, Krause et al. (2013) suggested that a specific type of stimulation may be either beneficial or unfavorable for local brain functioning, depending on whether it pushes or pulls the brain region toward or away from its optimal excitability level.

Cortical excitability can be quantified by the excitation/inhibition balance in a particular cortical region. This balance is thought to be determined by two key neurotransmitters: GABA, which has an inhibitory effect, and Glutamate, the brain’s main excitatory neurotransmitter (Petroff, 2002). Magnetic resonance spectroscopy (MRS) is a relatively novel method that allows for non-invasive, *in vivo* quantification of neurotransmitter levels such as GABA and Glutamate in a particular voxel in the human brain. Interestingly, MRS can thus be used to acquire individual Glutamate/GABA ratios that can be taken as a proxy for local cortical excitability in a specific target brain area of interest.

In line with the cortical excitability hypothesis, studies that combined MRS with tDCS in humans have related tDCS stimulation with both changes in GABA and Glutamate. More specifically, anodal stimulation over the motor cortex was shown to reduce resting-state GABA levels (Stagg et al., 2009), while cathodal stimulation in contrast reduced Glutamate levels (Clark et al., 2011). Although through different mechanisms, both types of stimulation may thus change the local excitation/inhibition balance and thereby critically alter neuronal functioning within the underlying cortex.

So far, most of the research applying both MRS and tDCS has been done in the motor domain and focused on the motor cortex. However, as effects of tDCS at the cellular level are not expected to be different for different parts the cortex, effects of tDCS on GABA and Glutamate should be similar for brain regions involved in higher-order cognitive functions, such as WM. In the current study, we aimed to investigate possible interactions between tDCS response and baseline cortical excitability further with regard to prefrontal tDCS and WM. More specifically, we examined if prefrontal cortical excitability levels (Glutamate/GABA ratios) determine behavioral effects of left DLPFC tDCS on verbal WM performance across individuals.

In an initial MRS session, we used 3T-MRS to measure GABA and Glutamate levels in the lDLPFC to determine baseline cortical excitability in this region in 20 healthy male subjects. Subsequently, in two stimulation sessions (separated by 1 week), we admitted once anodal and once cathodal tDCS over the lDLPFC (reference supraorbital in both cases, cf. Talsma et al., 2016). In both tDCS sessions, before, during and after stimulation, subjects performed a verbal WM task (the letter N-back task) to determine WM performance. The difficulty of this task was tailored to subjects’ individual WM updating capacity to allow for enough room for tDCS to increase or decrease WM performance, as well as to make the task equally challenging in all subjects.

Based on previous findings (Fregni et al., 2005; Ohn et al., 2008; Andrews et al., 2011; Lally et al., 2013; Talsma et al., 2016), we expected that anodal tDCS over the lDLPFC would improve WM accuracy in the majority of our subjects, resulting in a general improvement in WM performance compared to cathodal tDCS. Yet, in line with earlier reports, we also expected the effects of anodal stimulation to vary across subjects, with some subjects showing larger improvements after anodal prefrontal tDCS than others, and some perhaps showing decrements in WM performance. As the effects of cathodal stimulation in the cognitive domain are less conclusive (Jacobson et al., 2011), we expected no group-level effect of cathodal stimulation or a general decrement in performance.

We made two predictions with regard to the relation between baseline cortical excitability and the effect of stimulation. First, as anodal tDCS is associated with reducing GABA (Stagg et al., 2009), we expected a negative relationship between baseline cortical excitability and anodal tDCS-induced WM improvements. That is, we expected that subjects with lower baseline Glutamate/GABA ratios in lDLPFC (i.e., relatively higher baseline GABA concentrations) would show the biggest enhancements, as here anodal tDCS may help increase initial lower than optimal activation in this area. In contrast, as cathodal tDCS may specifically lower Glutamate levels (Clark et al., 2011), for cathodal tDCS, secondly, we expected a positive relationship, with cathodal stimulation being most beneficial in subjects with high baseline cortical excitability levels.

## Materials and Methods

### Participants

Twenty healthy, right-handed male participants participated in the study (Age range: 18 to 26, mean: 21.8, *SD*: 2.6). Female participants were excluded because cortical GABA concentrations have been reported to vary over the menstrual cycle (Harada et al., 2011; De Bondt et al., 2015). Subjects were recruited from a previous study sample for which we had already acquired 3T-MRS data (Talsma et al., submitted). They were screened for tDCS contra-indications (see Nitsche et al., 2008) and were paid for their participation in the form of course credit or with a monetary compensation. One subject did not complete the study because of excessive itching during stimulation (Age range remaining sample: 18 to 26, mean: 21.7, *SD*: 2.3). All procedures in this experiment were approved by the University of Amsterdam’s Ethical Committee.

### Procedure

Participants came to the lab for a total of four sessions, the first two of which were also part of a previous study (Talsma et al., submitted). In a first behavioral session, we determined working memory updating capacity (WMC) for each participant using an adaptive version of a verbal WM updating task (the letter N-back, see below for further details). In a second MRS-session, we used 3T-MRS to measure individual GABA and Glutamate concentrations in the left DLPFC [note: this data has previously been reported in Talsma et al. (submitted)].

In the third and fourth session, exclusive for this study, subjects came to the lab for two stimulation sessions at the same time of the day and spaced exactly 1 week apart. In one of the two stimulation sessions, subjects received anodal tDCS over the lDLPFC, while in the other session, they received cathodal stimulation (both 1 mA, 20 min) while performing a verbal WM updating task (the letter N-back). Order of stimulation type was counter-balanced between subjects. Notably, due to planning restrictions of the study, the first stimulation session took place on average 40 days after the MRS session (*SD*: 8.5, range: 29–67). However, high intra-subject stability of neurotransmitter levels have previously been reported over the course of 4 weeks (Bogner et al., 2010) and thus can be assumed to remain similar over time. Moreover, our recent study showed high consistency also over different activity ‘states’ (Talsma et al., submitted), indicating that the GABA and Glutamate concentrations measured with (3T-) MRS likely reflect relatively stable ‘traits’ that show consistency over time.

**FIGURE 1:**
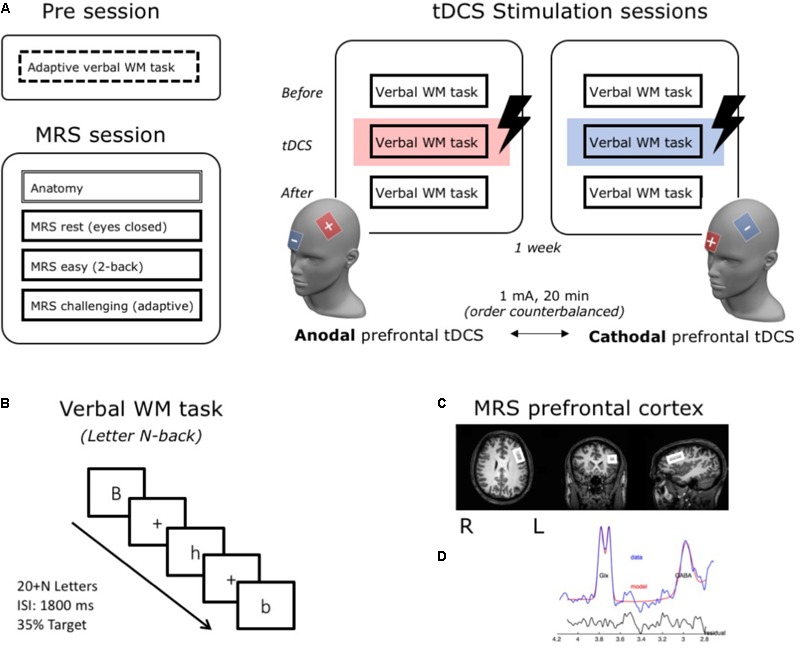
Schematic illustration of the research design and methods. Subjects came to the lab for a total of four sessions **(A)**. In a first behavioral Pre session, WM updating capacity (individual N) was determined for each participant using an adaptive version of the verbal WM updating task. In a second MRS-session, 3T-MRS was used to measure individual GABA and Glutamate concentrations in the left DLPFC under three conditions: rest, an easy and a challenging WM task. Notably, because we observed no differences between these three conditions [as reported in Talsma et al. (submitted)], for this study we averaged over all conditions and calculated Glutamate/GABA ratios to use as a measure for prefrontal cortical excitability. In two subsequent stimulation sessions, participants performed three blocks of a verbal WM task. During the second block, they received either anodal or cathodal stimulation over the lDLPFC (active electrode – F3, reference – above the right eye). **(B)** In the verbal WM task (letter N-back), a stream of letters was presented and participants were required to press a button if the current letter was the same as N stimuli before. In the adaptive version in the Pre session, the level of N was on-line adjusted to performance and gave us a measure for individual updating capacity for each subject (individual N). For the stimulation sessions, level of N consequently ranged between –1 and +2 around this individual updating capacity level to ensure a challenging task level for all subjects. **(C)** MRS-voxel location over the lDLPFC (size: 30 mm × 20 mm × 25 mm). **(D)** Modeling of the GABA and Glx (Glutamate + Glutamine) signal for a typical subject [output from the Gannet toolbox (www.gabamrs.com)]. In blue the edited spectrum is shown, overlaid in red is the model of best fit (using a simple Gaussian model) and the residual of these is shown in black.

In each of the two stimulation sessions, participants were seated comfortably behind a computer screen (at approximately 90 cm distance). Before the WM task started, rubber straps were put into place and the lDLPFC was localized in each participant (see below; cf. Talsma et al., 2016). This allowed for a fast placement of the electrodes right before the stimulation block, but prevented the sponges from drying out. After a brief practice session with feedback, subjects performed three blocks (±20 min each) of the letter N-back updating task (see for details below) (cf. Talsma et al., 2016). The first block of the task was administered before stimulation started and thus served as a baseline condition. The second block began 90 s after stimulation was started and ran throughout the entire stimulation time. The third block of the task was started after stimulation had ended. See **Figure 1A** for a schematic overview of the study design.

### Measuring WM Performance: The Letter N-Back Task

In all four sessions, the letter N-back task was used to measure WM performance (see also **Figure 1B**). In this task, a stream of letters is presented and subjects are asked to indicate if the currently presented letter is the same as the one presented N stimuli back. N is an integer and the value of N hence determines the difficulty level of the task: with higher levels of N, more stimuli have to be held in WM in sequential order, increasing WM load.

Because WM content has to be continuously updated, the letter N-back task is well suited to investigate WM updating performance (Jaeggi et al., 2010). Moreover, performance on this task has consistently been related to processing in the lDLPFC [e.g., see meta-analysis by Owen et al. (2005)]. Furthermore, although recent meta-analyses raise questions with regard to the reliability of anodal tDCS to lDLPFC to enhance WM performance (e.g., Dedoncker et al., 2016), we previously found that anodal lDLPFC tDCS enhanced accuracy on a very similar version of this letter N-back task (Talsma et al., 2016).

Presentation software (Neurobehavioural Systems, Inc.) was used to administer the letter N-back task. Black letters were presented (Arial, font size 72, letterset [“A,” “B,” “C,” “D,” “E,” “F,” “G,” “H,” “J,” “K’]) for 300 ms at the center of a white screen, followed by a 1500 ms inter-stimulus interval in which a fixation cross was displayed (Arial, font size 20). Of the presented letters, 37.50% were so-called targets (alternatingly 7 or 8 per run), i.e., a letter that was the same as the letter presented N trials back. Letters could be presented in upper or lower case and still classified as the same letter (i.e., a target). When presented with a target, subjects were required to press the space bar on the keyboard in front of them. In the Behavioral Session and MRS Session, participants performed an adaptive version of the N back task in which level of N was adjusted based on their task performance (see below). In the two subsequent tDCS sessions, participants performed the task at four different levels of N, which were chosen based on their performance in the first two sessions (see below).

Runs of the task always consisted of a stream of 20 + N stimuli each and were self-paced in the behavioral and stimulation sessions to allow the subject to take small breaks in between and enhance focus during the runs. The verbal N back task in the Behavioral session and every task block (before, during, and after) in the Stimulation Session consisted of 24 runs each. WM task set-up in the MRS-session was identical to the letter N-back task specifics as described above, except that because of the dimly lit nature of the scanning room in this case white letters were presented on a black background. Also, trials started automatically to ensure the task was performed for the entire time of the scan, resulting in 15 to 19 runs being performed per subject (depending on their individual level N).

#### Tailor Task Difficulty Using Individual WM Updating Capacity Scores

To ensure a challenging task level for all subjects, but also leave enough room for tDCS to improve or impair WM performance, we individually determined the level of N to be used in the two tDCS sessions for each subject based on their average WM updating capacity score. This score was determined using their performance on the adaptive version of the letter N-back task that was admitted in the behavioral and in the MRS-session.

In the adaptive letter N-back, level of N always started at 2, but was adjusted per run according to performance, with N incrementing one level after fewer than three errors (false alarms + misses) and decrementing one level after more than five errors (similar to Jaeggi et al., 2008). To determine WM updating capacity, we took the mean level N that subjects achieved in the last 21 runs of this task in the behavioral session and in the 12 runs in the MRS session, and averaged these scores. In both sessions, the first 3 runs were disregarded to allow some ramp-up time. As expected, in our sample, we observed a relatively large spread in the resulting individual WM updating capacity scores, with level N ranging between 2.7 and 6.1 (mean: 4.4, *SD*: 1.2).

The individual capacity scores were used to choose the levels of N for each subject separately in the stimulation sessions to ensure similar task difficulty for all subjects. Specifically, we determined *individual* N’s by rounding off WM updating capacity scores to the nearest integer. Across subjects, this resulted in individual N’s ranging between 3 and 6 (number of subjects per level – 3:5, 4:3, 5:6, 6:5). Then, in the stimulation sessions, level N’s ranged between *individual N* -1 and *individual N* +2. Thus, task level on the letter N-back ranged over four levels, which allowed for enough room to observe tDCS-related improvements as well as possible decrements in performance in every subject. In the stimulation sessions, each block of the task (before, during, and after stimulation) consisted of 24 runs, where N incremented twice over the different levels (individual N -1, individual N, individual N +1, individual N +2).

#### Determining WM Performance in the Stimulation Sessions

For each stimulation session separately, WM performance accuracy on the letter N-back task was operationalized by calculating A′ scores for each of the three blocks of the task (before, during, and after stimulation), averaged over the levels of N to obtain a reliable estimate of performance at the individual subject level (cf. Talsma et al., 2016). A′ is the non-parametric variant of signal detection theory’s d′ and takes into account both hits (correct responses) and false alarms (incorrect responses). In contrast to d′, A′ can account for situations in which participants do not show any false alarms, which may occur on easy task levels. A′ scores range from 0 to 1, in which 0 indicates chance performance and 1 perfect accuracy. A′ can be calculated from hit rate (H) and false alarm rate (F) with the following formula (Zhang and Mueller, 2005):

$$A\text{′}\;=\;\{\begin{array}{ll} \frac{3}{4}+\frac{H-F}{4}-F(1-H) & ifF\leq 0.5\leq H;\\ \frac{3}{4}+\frac{H-F}{4}-\frac{F}{4H} & ifF\leq H<0.5;\\ \frac{3}{4}+\frac{H-F}{4}-\frac{1-H}{4(1-F)} & if0.5<F\leq H. \end{array}$$

Additionally, to allow the investigation of possible speed-accuracy trade-offs, as well as to investigate possible stimulation effects on WM response speed, we calculated average reaction times over the correct responses for each block of the letter N-back task and each stimulation session separately.

### Measuring Prefrontal Cortical Excitability: 3T-MRS Data Acquisition and Analysis

In the MRS-session, for each subject we measured GABA and Glutamate levels in the lDLPFC (see also Talsma et al., submitted). Scanning was performed on a 3T Philips Achieva TX MRI scanner (Philips Healthcare) using an eight-channel head coil. According to individual anatomical landmarks as visible on an initial anatomical scan, the experimenter positioned the MRS voxel (30 mm × 20 mm × 25 mm) on the middle frontal gyrus and with the rear of the voxel anterior to the precentral sulcus (see also **Figure 1C**). Care was taken not to include cerebral spinal fluid (CSF) from the ventricles or the cortical surface.

We used a GABA-specific sequence of the Mescher-Garwood point-resolved spectroscopy (MEGA-PRESS) method (Waddell et al., 2007) to acquire Edited ^1^H J-difference spectra. The acquisition of this scan took approximately 12 min, during which 384 transients were collected (TE = 73 ms; TR = 2,000 ms). On the odd transients, a 15.64 ms sinc-center-editing pulse (64 Hz full width at half maximum) was applied in an interleaved manner at 1.9 and 4.6 ppm to excite GABA and suppress water respectively.

Neurotransmitter levels in the lDLPFC were measured three times in every subject: once at rest (eyes closed), once while they performed an easy WM task (letter 2-back) and once during a challenging WM task (adaptive letter N-back). Due to time constraints, the rest scan data of one subject could not be collected. In our previous study, we found that GABA and Glutamate levels did not differ between activity states [i.e., at rest vs. on-task (Talsma et al., submitted)]. Therefore, for the current study we averaged GABA and Glutamate concentrations across the different activity conditions to index individual neurotransmitter levels.

Spectral data were analyzed with the MATLAB-based package GANNET v2.1 (Edden et al., 2012^1^) as also described in Talsma et al. (submitted). Using the in-build options of the GannetLoad-function, the following processing steps were performed (in this order): time-domain frequency-and-phase correction using spectral correction, line broadening with an exponential apodization function, FFT, time averaging, frequency and phase correction based upon fitting of the Cho and Creatine signals, pairwise rejection of the data for which fitting parameters are greater than 3 SD from the mean, and finally, subtraction of the even from the odd transients to generate the edited difference spectrum. Note that in this edited difference spectrum, the GABA signal is contaminated by the macromolecule homocarnosine (Edden et al., 2012), a GABA derivative, and thus often referred to as GABA+.

Subsequently, using the GannetFit function of GANNET, GABA and Glx (the combined signal for Glutamate and Glutamine) functions were modeled to the data together (see also **Figure 1D**) and ratios relative to Creatine (Cr) were calculated (i.e., GABA+/Cr and Glx/Cr). Normalizing values to Creatine has been shown to reduce inter-subject variance as a result of differences in global signal strength, as well as differences stemming from tissue fractions in the scanned voxel (gray matter, white matter, and cerebrospinal), thus making coregistration, segmentation, and the calculation of CSF corrected values superfluous. Moreover, normalizing to Creatine has shown superior to normalizing to H_2_O with regard to intra-subject stability and therefore can be considered the most reliable measure for concentration estimates (Bogner et al., 2010).

Data of scans was excluded when the modelfit was poor (*N* = 2; corresponding to FitError > 15), and when the GABA or Glx-peak could not be confidently be determined (*N* = 1; GABA SNR < 3). Furthermore, in SPSS we identified outliers and excluded these from the data (*N* = 4, all values for GABA). Because previous analyses did not reveal differences between the three activity conditions, for the current study we averaged GABA (GABA+/Cr) and Glutamate (Glx/Cr) concentrations over the remaining scans per subject. Subsequently, a measure for cortical excitability was calculated by dividing Glutamate over GABA, resulting in a prefrontal Glutamate/GABA ratio for each subject.

For 13 subjects, all values could be used to determine individual GABA and Glutamate concentrations. However, as a result of the data exclusion as described above, we had to average over two of the three scans to determine GABA in four subjects, and for Glutamate in three subjects. For two participants, we could include only one scan to determine GABA. Similarly, for one last subject we determined Glutamate from one scan only. Resulting GABA concentrations (GABA+/Cr) ranged between 0.08 and 0.14 (mean: 0.12, *SD*: 0.02) and Glutamate concentrations (Glx/Cr) between 0.04 and 0.16 (mean: 0.11, *SD*: 0.03).

### Transcranial Direct Current Stimulation

Transcranial direct current stimulation was delivered with a battery-driven Eldith DC-stimulator (NeuroConn GmbH, Germany) using two 7 cm × 5 cm conductive electrodes. Electrodes were placed in saline-soaked sponges and held in place with rubber bands. In both sessions, after the baseline task block, one electrode was placed over the left DLPFC (F3 in the 10/20 system) and the other was placed over the right supra-orbitofrontal region (centered above the right eye pupil) (cf. e.g., Talsma et al., 2016), see **Figure 1A**). In the first stimulation session, the position of F3 was localized in each participant using an EEG cap (64 channels, Biosemi, Amsterdam, Netherlands). This position was marked on the scalp as well as measured relative to landmarks like the tip of the nose, the inion, and ears, to ensure identical electrode positioning in the second session. In both tDCS conditions, stimulation was applied for 20 min on 1 mA, once with the anodal electrode over the lDLPFC (i.e., anodal tDCS condition) and once the cathode (i.e., the cathodal tDCS condition). To reduce discomfort, in both conditions, the current was ramped up over 90 s and down over 90 s. The experimenter made sure that the resistance was always kept below 10 (range: 1.0 to 7.1, mean: 3.2, *SD*: 1.3). Both participant and experimenter were blind to the type of stimulation that was applied in each session.

Additionally, at the beginning and the end of each stimulation session, subjects filled out a questionnaire to assess physical sensations and register possible (negative) side effects of tDCS on mood and arousal levels. To investigate possible physical side effects of tDCS, at the end of each tDCS session, participants were asked to rate their experience on a five-item scale (namely “not,” “a little,” “somewhat,” “strongly,” and “very strongly”) of each of eight following sensations: itching, prickling, burning, pain, headache, fatigue, dizziness, and nausea. In addition, to assess mood and arousal levels, a Dutch translation of the short version of the Activation Deactivation Adjective Checklist (AD ACL) was used (Thayer, 1978), which requires participants to rate 20 items using a four-point scale (namely “definitely feel,” “feel slightly,” “do not really feel,” and “definitely do not feel”). Answers are scored on four subscales: energy (general activation), tiredness (general deactivation), tension (high preparatory arousal), and calmness (low preparatory arousal). The AD ACL has proven reliable and valid, showing high test–retest reliability for each of its subscales (all > 0.79; Thayer, 1978). The AD ACL was filled out pre- and post-stimulation in both tDCS sessions, and changes in mood and arousal were calculated for each session separately.

### Analytical Approach and Data Analysis

Firstly, we investigated the group-level effects of anodal and cathodal stimulation on WM performance. For this, we conducted a 2x3 repeated measures ANOVA on accuracy scores, with Stimulation type (Anodal vs. Cathodal) and the three blocks of the task (before, during, and after stimulation) as within-subject variables. We repeated this analysis, but with RT as the dependent variable. In case of significant effects, *post hoc* analyses were performed to further investigate findings and whenever appropriate Greenhouse–Geisser corrected values are reported. Additionally, to investigate whether order of the stimulation sessions or individual differences in WM updating capacity may have affected the effects of stimulation, we also reran the repeated measures ANOVA’s for both Accuracy and RTs, adding session order and individual WM capacity (individual N) separately as a covariate.

Secondly, next to determining group effects of tDCS, we tested our hypothesis that baseline lDLPFC cortical excitability levels may predict individual differences in the effect of anodal and cathodal stimulation on WM performance using correlation analyses. We previously observed the largest effects of tDCS not during, but after stimulation and on WM accuracy specifically (Talsma et al., 2016). Therefore, we quantified the tDCS effect on WM accuracy (A′) by subtracting baseline performance (i.e., in the first block of the task, before stimulation was applied) from performance in the block after stimulation, and divided this over baseline again to get a measure of relative improvement after tDCS per subject [i.e., (after-before)/before]. This was done separately for the anodal and cathodal stimulation session. Because of our relatively small sample, we ran Spearman rank correlations to determine the relationship between the MRS-measured prefrontal Glutamate/GABA ratios and these individual effects of anodal (cathodal) tDCS on WM performance.

Lastly, to examine possible non-specific physical or arousal effects of anodal and cathodal stimulation, we ran repeated-measures ANOVAs for each of the eight items on the tDCS side-effects questionnaire with Stimulation Type (anodal, cathodal) as a within-subject factor and Session Order as a covariate. To determine whether there was a difference in the effects of the two types of stimulation on arousal states or mood, scores on each of the four subscales of the AD ACL questionnaire were calculated before and after stimulation for each session separately and subsequently subtracted from each other to obtain a measure of the effect of each type of stimulation on arousal and mood. For each subscale separately, we then conducted a repeated-measures ANOVA with Stimulation Type (anodal, cathodal) as a within-subject factor and Session Order as a covariate, thus comparing changes in the resulting difference scores between the tDCS conditions. A Bonferroni correction was applied to account for multiple comparisons for both questionnaires separately, resulting in an alpha of 0.05/8 = 0.0063 for the physical side effects questionnaire and an alpha of 0.05/4 = 0.0125 for the Short Form AD ACL questionnaire.

All statistical analyses were conducted using the Statistical Package for the Social Sciences for Mac OS, Version 24 (IBM, Armonk, NY, United States). Furthermore, because of significant advantages over conventional statistics (Wagenmakers et al., 2017b), we additionally ran Bayesian analyses using the open-software package JASP^2^ (see also Wagenmakers et al., 2017a). Bayes factors will be reported, grading the intensity of evidence for the alternative hypothesis (Bf10), and values will be interpreted according to the corresponding classification scheme (see for elaboration Wagenmakers et al., 2017a): 1/30 < Bf < 1/10, Strong evidence for H0; 1/10 < Bf < 1/3, Moderate evidence for H0; 1/3 < Bf < 1, Anecdotal evidence for H0; Bf = 1, No evidence; 1 < Bf < 3, Anecdotal evidence for H1; 3 < Bf < 10, Moderate evidence for H1; 10 < Bf < 30, Strong evidence for H1.

## Results

### General WM Performance

In both stimulation sessions, all subjects showed good, but not ceiling level overall WM performance (mean A′: 0.84, *SD*: 0.038, range: 0.74–0.91; mean RT: 785, *SD* = 141, range: 590–1146). This indicates that our method to adapt task-levels according to individual WM updating capacity worked well.

Importantly, baseline performance did not differ between the two stimulation sessions, not in accuracy [*t*(18) = 0.504, *p* = 0.621, Bf = 0.266] or in reaction times [*t*(18) = 0.892, *p* = 0.384, Bf = 0.338]. Moreover, accuracy scores in this first block of the task ranged between 0.78 and 0.92 in the anodal (mean: 0.84, *SD*: 0.04) and between 0.79 and 0.87 in the cathodal stimulation session (mean: 0.84, *SD*: 0.03), indicating enough room to improve (as well as possibly deteriorate) as a function of tDCS stimulation in both stimulation sessions. Please see **Table 1** for the mean and *SD* of both accuracy and RTs over the different task blocks for both stimulation conditions.

**Table 1:** Mean and standard deviations shown separately for accuracy (A′) and RTs on the verbal WM updating task in the two stimulation sessions (*N* = 19), split out for the three different blocks of the task and the anodal and cathodal tDCS stimulation condition.

	Anodal tDCS		Cathodal tDCS	
	Accuracy Mean (*SD*)	RTs Mean (*SD*)	Accuracy Mean (*SD*)	RTs Mean (*SD*)
Before	0.84 (*0.04*)	674 (*113*)	0.84 (*0.03*)	663 (*114*)
tDCS	0.85 (*0.05*)	843 (*169*)	0.84 (*0.05*)	828 (*130*)
After	0.85 (*0.05*)	856 (*193*)	0.84 (*0.08*)	845 (*170*)

### Group-Level Effects of Anodal and Cathodal tDCS on WM Performance

We first investigated the effect of anodal and cathodal stimulation on WM performance at the group level by running a 2 (Stimulation Type) × 3 (Block) repeated measure ANOVA for Accuracy and RTs separately. See **Table 1** for an overview of mean accuracy and RT per Stimulation type and Block.

Overall, accuracy did not change over the different blocks of the task [Main effect Block: *F*(2,36) = 0.575, *p* = 0.499, Bf = 0.09], nor did it significantly differ between anodal and cathodal stimulation [Main effect Stimulation Type: *F*(1,18) = 0.007, *p* = 0.933, Bf = 0.47]. Moreover, the critical interaction effect between Stimulation Type and Block was non-significant indicating that anodal and cathodal did not differentially affect WM accuracy [Interaction Type ^∗^ Block: *F*(2,36) = 0.560, *p* = 0.523]. Furthermore, a Bayesian model including the two main effects (Bf = 0.05) and one which additionally included the interaction (Bf = 0.01), both showed more evidence for the null-hypothesis. Thus, anodal and cathodal stimulation did not (differentially) affect verbal WM accuracy.

As to Reaction Times, response times did not significantly differ between the anodal and cathodal session [Main effect Stimulation Type: *F*(1,18) = 0.996, *p* = 0.331, Bf = 0.231]. Although in both sessions, subjects’ responses became slower over time [Main effect Block: *F*(2,36) = 92.053, *p* = 0.000, Bf = 6.01 ^∗^ 10ˆ22], the extent to which responses became slower over time did not differ between the stimulation conditions [Interaction Block ^∗^ Stimulation Type: *F*(2,36) = 0.025, *p* = 0.975]. These findings indicate that anodal and cathodal stimulation did not have a differential effect on WM response times. Indeed, our Bayesian analyses showed extreme evidence for the alternative hypothesis of no effect, both in a model that included both main effects (Bf = 2.13^∗^10ˆ22) and in one which additionally included the interaction between the two (Bf = 3.17^∗^10ˆ21). Moreover, a direct comparison between these two models critically shows moderate evidence in favor of a model in which the interaction is not included (Bf = 0.15). Thus, we found no effect of stimulation type on RT either. The observed slowing in RT likely reflects a general fatigue effect in both conditions.

To control for possible confounding effects of session order and individual differences in WM updating capacity, we ran the analyses on both accuracy and RT again adding these as covariates. This did not change the pattern of findings. However, comparing accuracy in the first block of the task in both sessions directly, showed a trend-level difference [*t*(18) = -2.071, *p* = 0.052, Bf = 1,352] with performance being higher in the second (mean A′: 0.85, *SD*: 0.035, range: 0.78–0.92) compared to the first session (mean A′: 0.83, *SD*: 0.033, range: 0.78–0.88). This provides some indication for a practice effect in that subjects seemed to get better over the stimulation sessions.

**FIGURE 2:**
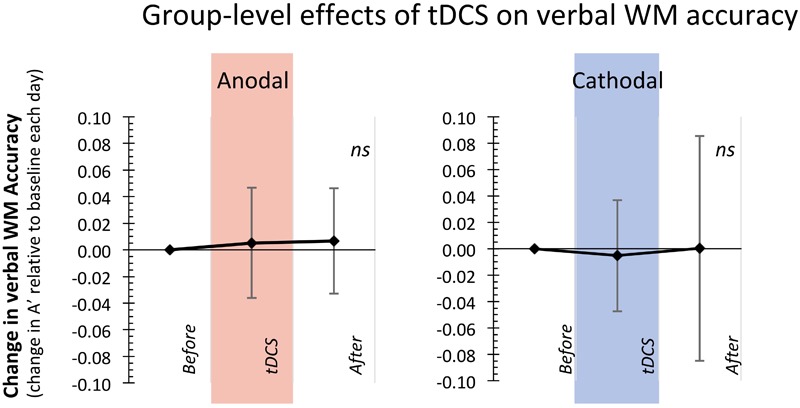
Group-level analyses showed that anodal and cathodal prefrontal tDCS stimulation did not consistently affect WM performance. Displayed here is the change in Accuracy (A′) for the blocks of the task during and after tDCS stimulation relative to the baseline block of that day (error bars represent Standard Deviations from the mean).

Together, these findings indicate that at the group-level, neither type of tDCS stimulation over the lDLPFC (anodal nor cathodal) consistently altered accuracy (see also **Figure 2**) or reaction times on the verbal WM updating task. Thus, in contrast to our expectations, in the current study we do not replicate our previous group-level findings (Talsma et al., 2016) that anodal stimulation over the lDLPFC concurrent with a verbal WM updating task improves WM accuracy.

### Does lDLPFC Cortical Excitability Levels Predict the Effect of tDCS on WM?

To answer our main research question, we next examined if individual differences in the effects of anodal and cathodal tDCS on WM performance across subjects can be predicted by baseline prefrontal cortical excitability levels, as measured with 3T-MRS. For this, we first quantified the effect of anodal and cathodal stimulation on WM accuracy for every subject as a relative change to baseline per session [(After–Before)/Before]. Eyeballing our data, we found that about half of our subjects improved in the block after anodal tDCS (*n* = 11), while the other half showed decreased WM accuracy after stimulation compared to before (*n* = 8). Similarly, in the cathodal stimulation session, accuracy improved after stimulation in approximately half of our subjects (*n* = 10), while it deteriorated in the other subjects (*n* = 9).

**FIGURE 3:**
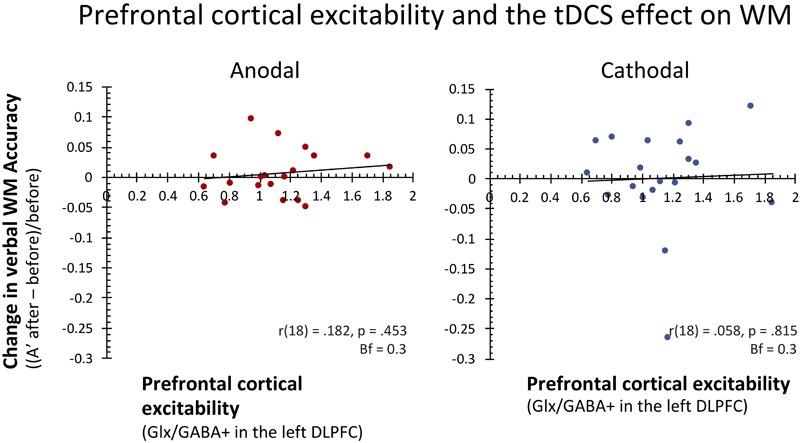
In contrast to our expectations, baseline prefrontal cortical excitability (Glutamate/GABA ratios) did not predict individual differences in the extent and direction of the effects of anodal and cathodal tDCS on WM. As can be seen in these scatterplots, in both stimulation conditions about half the subjects showed improved verbal WM after stimulation, while the other half showed worsened performance. Pearson correlation coefficients and two-tailed *p* statistics are reported, as well as Bayes factors (Bf10).

To test our main hypothesis, we subsequently correlated prefrontal cortical excitability levels (Glutamate/GABA ratios) with these behavioral effects across subjects. In contrast to our expectations, cortical excitability levels in lDLPFC did not predict the effect of anodal tDCS on WM performance [*r*(18) = 0.182, *p* = 0.453, Bf = 0.32]. Similarly, prefrontal cortical excitability also did not predict the effect of cathodal prefrontal tDCS stimulation on verbal WM [*r*(18) = 0.058, *p* = 0.815, Bf = 0.29] Removing one subject that showed extreme deterioration in the cathodal stimulation condition (>3 *SD* from the mean) did not change this result [*r*(17) = 0.091, *p* = 0.720, Bf = 0.31]. In both cases, Bayesian statistics indicated moderate evidence for the lack of a relation between baseline cortical excitability and individual differences in the effect of tDCS on WM accuracy (see also **Figure 3**). Furthermore, *post hoc* additional analyses that related GABA (GABA+/Cr) and Glutamate (Glx/Cr) separately to the effects of anodal and cathodal tDCS were not significant either (all *p*’s > 0.375; all Bf’s < 0.42).

In conclusion, even though we observed large variability in both the extent and direction of the effects of anodal and cathodal on WM performance, prefrontal cortical excitability did not predict the effect of anodal or cathodal lDLPFC stimulation on WM performance across subjects.

### Questionnaires

On the tDCS side effects questionnaire, no differences were reported between the anodal and cathodal stimulation condition for any of the possible physical sensations (all uncorrected *p*’s > 0.25, Bf’s < 0.6), and no significant interactions were found with session order (all uncorrected *p*’s > 0.12, Bf’s < 0.6). Overall, subjects reported to have felt ‘somewhat’ of an itching (mean: 1.87, *SD*: 1.16) and prickling (mean: 1.84, *SD*: 1.16) sensation, and experienced ‘a little’ of a burning (mean: 1.25, *SD*: 1.12) and a feeling of tiredness (mean: 1.43, *SD*: 1.21). However, importantly, they did not report general feelings of pain (mean: 0.24, *SD*: 0.49), headaches (mean: 0.37, *SD*: 0.65), dizziness (mean: 0.22, *SD*: 0.47), or nausea (mean: 0.03, *SD*: 0.12). On an individual level, the following number of subjects reported perceptions for the following sensations in one or both stimulation sessions: itching: *N* = 19, prickling *N* = 17, burning *N* = 12, pain *N* = 7, headache *N* = 7, fatigue *N* = 16, dizziness *N* = 4, and nausea *N* = 1.

Similarly, for the mood and arousal questionnaire, traditional statistics revealed that subjects reported equal changes for the anodal and cathodal stimulation condition on all subscales (all uncorrected *p*’s > 0.17), independent of the order in which they received each type of stimulation (all uncorrected *p*’s > 0.24). However, Bayesian statistics indicated that there is strong evidence for a difference in change in the subscale energy between the stimulation conditions [Bf = 11; unrelated to session order (Bf = 0.5)], but not any of the other subscales (Main effects Stimulation Type: Bf’s < 0.5, Interactions Stimulation Type ^∗^ Session Order: Bf’s < 0.24). The change in the level of energy was on average 0.68 (*SD*: 2.52) in the anodal and -2.26 (*SD*: 2.82) in the cathodal stimulation condition. Overall, subjects reported lower levels of energy (mean: 1.79, *SD*: 2.67), higher levels of tiredness (mean: -2.21, *SD*: 2.38) but no substantial differences in feelings of tension (mean: 0.45, *SD*: 1.97) or calmness (mean: -0.21, *SD*: 1.39) at the end compared to the beginning of the stimulation session.

## Discussion

In the current study, we aimed to investigate if baseline cortical excitability can explain individual differences in how tDCS affects cognitive functioning. Specifically, we tested if prefrontal cortical excitability levels, as indexed by 3T-MRS measured Glutamate/GABA ratios, can predict the extent to which anodal or cathodal prefrontal tDCS stimulation improves or impairs verbal WM performance across subjects.

Replicating previous observations of large individual variability in tDCS effects on WM performance (Berryhill and Jones, 2012; London and Slagter, 2015; Talsma et al., 2016), for both types of stimulation, about half of the participants showed improved verbal WM updating accuracy after stimulation, while the other half showed detriments. Yet, in contrast to our main expectations, baseline prefrontal cortex excitability did not predict the effects of anodal or cathodal tDCS on WM functioning across subjects. Moreover, in contrast to earlier studies (e.g., Fregni et al., 2005; Ohn et al., 2008; Andrews et al., 2011; Lally et al., 2013; Talsma et al., 2016), at the group-level, neither anodal nor cathodal stimulation affected WM performance.

Since we used the exact same stimulation parameters and a very similar verbal WM updating task and task design as in a previous study (Talsma et al., 2016), this latter finding is surprising. An important difference between the two studies that may have caused this is that in the previous one we excluded task ceiling level performers to ensure a homogenous subject population, in the current study we did not preselect subjects. Moreover, in our previous study, all subjects performed the task at levels 3 and 4, while here we adjusted level of N to ensure a similar level of task difficulty in each subject. It is also possible that the higher levels of N (N +2 above WM capacity) included in the current study were relatively challenging and caused our subjects to fatigue more quickly than in our previous study. This may have countered any beneficial effects of tDCS at the group level, and prevented us from replicating our previous finding. However, accuracy scores in the current study (mean A′: 0.84, *SD*: 0.038, range: 0.74–0.91) were very similar to accuracy scores observed in our previous study (First session: mean A′: 0.85, *SD*: 0.08, range: 0.72–0.96), suggesting that task difficulty was matched across studies (Talsma et al., 2016). It should also be pointed out that participants in both studies had equal amounts of practice on the task prior to stimulation (about 40 min in both studies). Thus, differences in practice also cannot explain the discrepancy in findings. Nevertheless, at the same time, these null findings add to the growing number of reports that the relation between anodal prefrontal stimulation and WM improvements is not as consistent as initially assumed (e.g., see the meta-analyses by Bennabi et al., 2014; Brunoni and Vanderhasselt, 2014; Dedoncker et al., 2016; Hill et al., 2016; Mancuso et al., 2016). Given the also noted variability in individual tDCS response, especially in smaller subject samples, group-level conclusions may be substantially affected by the specific selection of subjects within the sample, thereby creating inconsistencies in conclusions across the field.

These findings point out the importance of looking at why the effects of tDCS vary so substantially across subjects. In the current study, we tested the hypothesis that baseline cortical excitability levels (partly) determine the effect of tDCS on WM performance. However, in contrast to this notion, we found no evidence that baseline prefrontal cortical excitability levels predicted individual differences in the effects of anodal and cathodal prefrontal stimulation on WM. Being a pioneering report in this regard, below we will discuss some limitations of the current study and suggest directions for future research necessary to confidently determine the absence or presence of this relationship between baseline cortical excitability and tDCS effect.

A first limitation of the current study is the use of 3T-MRS to quantify Glutamate and GABA concentrations to calculate prefrontal cortical excitability levels. In the past decade, 3T-MRS has repeatedly been used to *in vivo* measure concentrations of the main inhibitory (GABA) and excitatory (Glutamate) neurotransmitters in the human brain, and to relate these neurotransmitter concentrations to individual differences in behavior (e.g., Yoon et al., 2016; see for an overview also Duncan, 2013). However, in a recent study (Talsma et al., submitted) with a larger subject sample than earlier studies, we failed to replicate two of these previously reported relations (Edden et al., 2009; Yoon et al., 2016). Specifically, prefrontal GABA did not predict WM updating, updating capacity or WM maintenance, nor did occipital GABA predict visual discrimination performance. These findings challenge the idea that 3T-MRS provides a measure that is sensitive enough to adequately quantify Glutamate and GABA concentrations predictive of cortical functioning and behavior. If so, 3T-MRS may also fail to provide the sensitivity that is necessary to successfully investigate the role of baseline cortical excitability in the effects of tDCS on behavioral performance.

A pivotal direction for future studies that aim to investigate the relationship between cortical excitability and tDCS effects may be to use 7T-MRS, which has two important advantages over 3T-MRS. Firstly, increased spectral resolution at higher magnetic field strengths enables better discrimination, and thereby quantification of the two neurotransmitters critical for the cortical excitation/inhibition balance: Glutamate (independent from Glutamine (Lally et al., 2016)) and GABA (uncontaminated by macromolecules (Chen et al., 2017)). Secondly, because of the better signal-to-noise ratios (Lally et al., 2016), smaller sized MRS voxels can be used, which may substantially enhance the spatial precision of the brain area for which one aims to determine cortical excitability.

Indeed, another factor that may have obscured a relationship between prefrontal cortical excitability and tDCS effects is the fact that the region of lateral prefrontal cortex that is considered critical to WM functioning (Owen et al., 2005) is much smaller than the voxel area that we need with current 3T- MRS to obtain a good enough signal. Placing a relatively large voxel over an actually much smaller region of interest may ‘delute’ the measure significantly, thereby reducing its sensitivity for regionally specific concentrations. Using functional MRI to localize the part of the left DLPFC that is involved in WM functioning in every subject individually and subsequently placing a smaller (7-T) MRS voxel over this area, could hence also substantially improve sensitivity of the measure of cortical excitability and thus be important to successfully study its relation to tDCS effects on behavior.

Lastly, three additional factors may have played a role in our findings. Firstly, in the absence of a systematic effect of tDCS at the group level, it is possible that our individual tDCS effects did not (solely) capture effects specific to tDCS, but (also) reflected non-specific effects related to time on task, such as changes in fatigue level, practice, or learning, or regression to the mean. Indeed, we found a trend-level difference in baseline accuracy (the block before stimulation) between the first and second stimulation session (averaged over both stimulation conditions). As we did not include a no-stimulation control group, it is difficult to determine the possible contributions of these possible confounding factors. Secondly, although levels of GABA and Glutamate are thought to be relatively consistent over time (Bogner et al., 2010), measuring these neurotransmitters closer in time to the actual stimulation than in the current study may enhance its sensitivity and thus yield more positive results. Lastly, next to cortical excitability, in conventional non-individualized tDCS set-ups, other factors may determine the effectiveness of tDCS to an even larger extent, such as anatomical differences affecting current flow (Kim et al., 2014) or the distance between the functional cortical region and the stimulation electrodes. Although the low spatial accuracy of conventional tDCS set-ups heightens the chance that the lDLPFC target area is affected at least to some extent in all participants, the exact amount of current that reaches the targeted cortical neurons in each subject is unknown but may greatly vary between individuals. These inaccuracies may mask contributions of more subtle factors such as delicate interactions of tDCS with the baseline cortical excitation/inhibition balance of the area. Therefore, developing more individualized stimulation protocols, for example that include an fMRI localizer, that allow for a more precise deliverance of a specific amount of current to the (individually localized) target brain area in every subject may thus be a critical next step before we can further investigate the role of baseline cortical excitability in determining the effect of anodal and cathodal tDCS on cognitive performance.

## Conclusion

To our knowledge, the current study is the first to test the hypothesis that baseline cortical excitability levels critically determine the effects of tDCS on cognitive functioning. Although we observed large individual differences in tDCS response (but no group-level effect of tDCS), baseline prefrontal cortical excitability levels did not predict which subjects improved and which actually deteriorated after anodal or cathodal stimulation. However, being a pioneering study, these findings should be interpreted with care and should first and foremost serve to direct the design of future studies in this field. Hopefully, this will eventually lead to a better understanding of tDCS and how it may improve WM. This knowledge is not only essential to help resolve current inconsistencies in the field, but also to ensure the practical application of tDCS to enhance WM functioning not just in some, but in all individuals.

## Ethics Statement

The Faculty Ethics Review Board of the Faculty of Social and Behavioural Sciences of the University of Amsterdam approved the study. All subjects provided written informed consent.

## Author Contributions

LT and HS designed the study. JB, JH (in equal amount), and LT collected the data. LT performed the data analysis and interpretation in consultation with HS. LT drafted the manuscript. HS, JB, and JH provided critical revisions.

## Conflict of Interest Statement

The authors declare that the research was conducted in the absence of any commercial or financial relationships that could be construed as a potential conflict of interest.

## Footnotes

1 http://www.gabamrs.com

2 http://www.jasp-stats.org
